# Targeted Therapy: A New Approach for the Treatment of Locally Advanced Oropharyngeal Cancer

**Published:** 2012

**Authors:** L.Z. Velsher, A.A. Kosmynin, M.Yu. Byakhov, T.K. Duditskaya, D.N. Reshetov

**Affiliations:** Moscow State Medical and Dental University; Cancer Center JSC “RZD”, Moscow

**Keywords:** oropharyngeal cancers, targeted therapy, quality of life, gefitinib

## Abstract

Presented herein is a clinical study comprising 48 patients (42 men and 6 women) of working age (40–70 years), all of whom are suffering from locally advanced oropharyngeal cancer. A modern approach is applied to treat these patients,*i.e.*, neoadjuvant targeted therapy, taking into account the biological profile of the tumor. The use of gefitinib causes an antitumor effect in 90.5% of cases as opposed to 56.5% when no drug is applied.

## INTRODUCTION

Malignant tumors of the head and neck account for 20% of overall incidences of cancer. Squamous-cell carcinoma of the head and neck (SCCHN) is one of the most frequently observed types among other malignant tumors, the number of cases exceeding 600,000 annually [1]. In Russia, more than 80,000 patients with the pathology are registered every year; 3.5% of them are patients suffering from cancer of the mouth and throat. In the territory of the Russian Federation, the incidence of oropharyngeal cancers increased from 24.7 to 29.6 per 100,000 for the period from 1997 to 2007. In more than 70% of the cases, patients seek medical care when the disease is advanced (stages III, IV); in these stages, radical surgery is either impossible or severely restricted [1, 2]. The percentage of advanced oropharyngeal cancer cases has risen from 26.4 to 31.2%; the mortality rate from this is 15.6% [3]. The severity and urgency of this problem is beyond question, since the problem affects people of working age.

Using surgical intervention for patients of this group involves performing extended and combined surgeries, which have a mutilating effect, thereby significantly impairing the quality of life. Radiation therapy, either in combination with surgical treatment or alone at high doses of radiation, causes the development of severe complications (xerostomia, dysphagia, mucositis, etc.), which significantly limit its application, and impair psychosocial adaptation and rehabilitation of the patients.

In addition, during combined therapy, local recurrence occurs in 10–30% of patients with SCCHN, including those with histologically normal resection margins; the latter indicates a probable subclinical systemic extension of the tumor, occurring even prior to the stage of generalization. In this context, the complex approach plays an increasingly important role not only in surgery and radiation therapy, but in medicamental treatment as well; in other words, it is a systemic action on tumor cells.

For a significant period of time, drug therapy against squamous-cell oropharyngeal cancer has been applied for palliative purposes in the inoperable cases of advanced, frequently recurrent cancers that are characterized by the presence of distant metastases.

All current regimens of polychemotherapy, in which platinum-based or 5-fluorouracil (5 FU) drugs are used, provide an objective response rate of 57–80%; while the use of taxanes provides up to 36–40%. The overall life expectancy of patients does not increase in both the aforementioned cases [4–9]. The low response of tumors forces researchers to seek new approaches for systemic treatment.

The recent period in the history of anticancer therapy began in the mid-1990s, although its foundations were laid as a result of the achievements in fundamental biology in the last two decades [10]. In those studies, the molecular mechanisms of regulation of cellular proliferation and differentiation were revealed; the latter enabled the development of drugs acting in a completely novel way. [11].

**Fig. 1:**
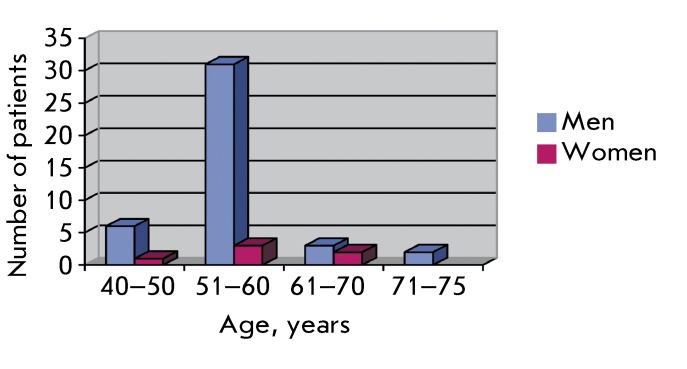
Distribution of patients with oropharyngeal cancer by age.

In contrast to classic cytostatic drugs, whose action is based on disturbing the cell cycle, the drugs for targeted therapy affect only particular molecular targets, thereby blocking the earlier stages of carcinogenesis. There are drugs of the above-mentioned type that can be applied for the treatment of squamous-cell oropharyngeal cancer [2, 10, 12–14].

The main target in the case of SCCHN is the epidermal growth factor receptor (EGFR). The hyperexpression of epidermal growth factor receptors is noted in approximately 90–100% of cases of squamous-cell oropharyngeal cancer and is associated with the worst prognosis of the disease, a low differentiation of the tumor, and a decrease in the total and recurrence-free survival rates [15–17].

In 2004, three research groups published data indicating that mutations in the EGFR tyrosine kinase domain increase the response of tumors to the following EGFR tyrosine kinase inhibitors: gefitinib and erlotinib [12, 16, 18]. The majority of mutations found in the *EGFR* gene are either deletions in exon 19 (29 from 56.52% of cases), which lead to the loss of four amino-acid residues (leucine, arginine, glutamic acid, and alanine) in a protein molecule, or point mutations in exon 21 causing the replacement of leucine in position 858 by arginine (20 from 56.36% of cases) [12]. The presence of mutations in the *EGFR* gene is an important predictor of the probability of a more favorable outcome in gefitinib therapy. The high effectiveness of gefitinib was confirmed for the case of non-small cell lung cancer with a mutation in the *EGFR* gene: the objective response rate was 84.6% [19–21]. Since hyperexpression of EGFRs is observed in more than 80% of malignant tumors of the head and neck [22], we began studying the effectiveness of combined cisplatin, 5-fluorouracile and gefitinib (Iressa) in patients suffering from advanced (stages III, IV) squamous-cell oropharyngeal cancer with a mutation in *EGFR.*

Gefitinib (Iressa) was one of the first tyrosine kinase inhibitors introduced into clinical practice. According to its chemical structure, this drug is a derivative of anilinoquinazoline. Gifitinib selectively and reversibly binds to the ATP-binding site of the EGFR tyrosine kinase domain, thereby blocking its tyrosine kinase activity, *i.e.* , its ability to phosphorylate the signal proteins found after this site; the latter leads to the inhibition of proliferative signals [23, 24]. Gefitinib induces an increase in the level of the cyclin-dependent kinase p27 inhibitor in the cell, in turn causing a delay of the cell cycle in G1. Active studies of gefitinib are being performed within international clinical trials. In the Phase II clinical trial, application of gefitinib in 52 patients with recurrent/metastatic SCCHN allowed to achieve an objective response in 10.6% of them and to attain a level of disease control in 53%. Half of the patient cohort received gefitinib as plan B therapy. Thus, the median progression-free survival and overall survival were 3.4 and 8.1 months, respectively. The only clinically significant side effect observed was diarrhea [25].

## EXPERIMENTAL

For the period from March 2009 to April 2011, 48 patients (42 men, 6 women) aged 40–75 years, a mean age of 57 years, were treated.

The diagram presented in *Fig. 1* clearly demonstrates that the bulk of the patients are men of working age. Oropharyngeal cancer is seven times more common in men than in women.

**Fig. 2:**
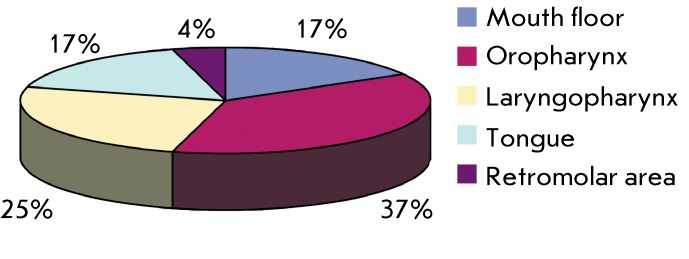
Distribution of patients with oropharyngeal cancer by the localization of the primary tumor (%).

Within the patients, the tumor was distributed as follows ( *Fig. 2* ): the mouth floor in 8 (17%); the oropharynx in 18 (37%); the laryngopharynx in 12 (25%); the mobile part of the tongue in 8 (17%); and the retromolar area in 2 (4%). In the diagram, it can be clearly seen that oropharyngeal and laryngopharyngeal cancers prevail, while cancers of the mobile part of the tongue and of the retromolar area are less common.

The area of tumor involvement before the beginning of therapy was assessed by clinical examination of the lesion area, along with computed tomography and ultrasonic examination of regional lymph nodes. 

Prior to therapy, the biological profile of the tumor, *i.e.* the expression of epidermal growth factor receptors and the presence of mutations in the *EGFR* gene, was determined in all patients. Mutations in the *EGFR* gene were revealed by polymerase chain reaction (PCR), and the expression of EGFRs was ascertained via the immunohistochemical method. Allele-specific PCR with primers specific to the L858R mutation in the *EGFR* gene was carried out on the DNA from paraffin blocks with an established tumor. The wild-type *EGFR* gene undergoes amplification, accompanied by an increase in *C* t by 7–10 cycles under the same conditions, thus enabling the above-mentioned gene to be distinguished from the mutant.

**Table:** Comparative analysis of the effectiveness of therapy in patients suffering from squamous-cell carcinoma of the head and neck, with and without the application of gefitinib

Group	Number of patients	Objective effect				Mutated* EGFR*	
		Complete response	Partial response	Stabilization	Progression	Yes	No
First (studied)	21	7 (33.3%)	9 (42.9%)	3 (14.3%)	2 (9.5%)	2 (4.5%)	20 (45.5%)
Second (control)	23	_	13 (56.5%)	_	10 (43.5%)	1 (2.3%)	21 (47.7%)
In total	100%					3 (6.8%)	41 (93.2%)

Naive patients suffering from locally advanced squamous-cell oropharyngeal cancer (stages III, IV) were randomly divided into two groups:

The first group (studied) received cisplatin (100 mg/m ^2^ , via intravenous administration, on the first day), 5-fluorouracil (500 mg/m ^2^ , via intravenous administration, from the first to the fifth day) (four cycles with intervals of 21 days); and gefitinib (Iressa) (250 mg per os, daily, for 16 weeks).

The second group (control) received cisplatin (100 mg/m ^2^ , via intravenous administration, on the first day) and 5-fluorouracil (500 mg/m ^2^ , via intravenous administration, from the first to the fifth day) (four cycles with intervals of 21 days).

After four cycles, the tumor response was assessed clinically and in accordance with the RECIST criteria.

In the second stage of complex therapy, the patients in whom complete resorption of the tumor was achieved were treated with radiation therapy in accordance with the radical program: the primary tumor was irradiated at a total dose of 60–70 Gy, and the regional lymph nodes – at a total dose of 30–40 Gy. The patients with partial regression and stabilization of the tumor process underwent preoperational radiation therapy at a total dose of 30–40 Gy, followed by surgery.

## RESULTS AND DISCUSSION

The effect of the therapy was assessed in 44 patients who went through a complete course of treatment. For four patients (16.7%), it was necessary to interrupt the treatment due to the appearance of toxic effects: in two (8.3%) of them, nephrotoxicity (stage III-IV) appeared; in the other two (8.3%), hematologic toxicity (stage IV) was observed. Hyperexpression of EGFRs was revealed in all 48 patients (100%). However, mutations in the *EGFR* were found in only three (6.8%).

A comparative analysis of the results of the therapies in both groups ( *Table* ) revealed that a complete clinical regression of the tumor was achieved in seven (33.3%) people from the group of 21 examined patients, and a mutation in the *EGFR* gene was found in two (4.5%) of those seven; in nine (42.9%) cases, a partial response (regression of the tumor of up to 85%) was observed; in three (14.3%) patients, stabilization of the tumor process (a decrease in the tumor by 18–20%) was detected; and in two (9.5%) patients, tumor progression was noted. In the control group of patients receiving only standard chemotherapy, a partial response (regression of the tumor of up to 57%) was observed in 13 (56.3%) of the 23 patients, while in 10 (43.5%) patients, the tumor continued to grow. 

## CONCLUSIONS

A significant increase was recorded in the effectiveness of the therapy in people suffering from oropharyngeal cancer by applying gefitinib, a drug used in targeted therapy. The effect of gefitinib is most pronounced when there are mutations in the *EGFR* gene. When therapy including gefitinib was used, the clinical tumor response was achieved in 90.5% of patients, which was twice higher than in the case of chemotherapy alone, 56.5%; and in 33.3% of cases, the result was achieved without surgery. The combination of targeted therapy with standard chemotherapy allows to increase the effectiveness of the therapy and to improve the prognosis for the disease. The results obtained in this work show the significant potential held by the application of this therapy scheme in conservative stages of treatment (chemoradiation therapy) during the early stages of the tumor; owing to this, organ-preserving complex treatment of patients suffering from squamous-cell oropharyngeal cancer may become possible. Clinical studies of the effectiveness of targeted drugs (erlotinib, gefitinib, cetuximab), applied in combination with chemoradiation therapy against squamous-cell carcinoma of the head and neck with a mutation in the *EGFR* gene, continue around the world. Altogether, the results of these studies will open up new opportunities for the treatment of the types of patients detailed above, improving their quality of life and enabling the performance of organ-preserving operations. 

## Abbreviations

SCCHN: squamous-cell carcinoma of the head and neck
EGFR: epidermal growth factor receptor
